# Depressed patients treated by homeopaths: a randomised controlled trial using the “cohort multiple randomised controlled trial” (cmRCT) design

**DOI:** 10.1186/s13063-017-2040-2

**Published:** 2017-06-30

**Authors:** Petter Viksveen, Clare Relton, Jon Nicholl

**Affiliations:** 1 The Department of Health Studies, The University of Stavanger, Kjell Arholms hus, Kjell Arholms gate 39, 4021 Stavanger, Norway; 2 The University of Sheffield, Faculty of Medicine, Dentistry and Health, School of Health and Related Research, Regent Court, 30 Regent Street, Sheffield, S1 4DA UK

**Keywords:** Randomised controlled trial, Depression, Anxiety, Homeopathy, Treatment by homeopaths

## Abstract

**Background:**

Despite controversy regarding homeopathy, some patients consult homeopaths for depression. Evidence is required to determine whether this is an effective, acceptable and safe intervention for these patients.

**Methods:**

A pragmatic trial using the “cohort multiple randomised controlled trial” design was used to test the effectiveness of adjunctive treatment by homeopaths compared to usual care alone, over a period of 12 months in patients with self-reported depression. One third of patients were randomly selected for an offer of treatment provided by a homeopath. The primary outcome measure was the Patient Health Questionnaire (PHQ-9) at 6 months. Secondary outcomes included depression scores at 12 months; and the Generalised Anxiety Disorder (GAD-7) outcome at 6 and 12 months.

**Results:**

The trial over-recruited by 17% with a total of 566 patients. Forty percent took up the offer and received treatment. An intention-to-treat analysis of the offer group at 6 months reported a 1.4-point lower mean depression score than the no offer group (95% CI 0.2, 2.5, *p* = 0.019), with a small standardized treatment effect size (*d* = 0.30). Using instrumental variables analysis, a moderate treatment effect size in favour of those treated was found (*d* = 0.57) with a between group difference of 2.6 points (95% CI 0.5, 4.7, *p* = 0.018). Results were maintained at 12 months. Secondary analyses showed similar results. Similar results were found for anxiety (GAD-7). No evidence suggested any important risk involved with the intervention.

**Conclusion:**

This trial provides preliminary support for both the acceptability and the effectiveness of treatment by a homeopath for patients with self-reported depression. Our results provide support for further pragmatic research to provide more precise estimates of treatment effect.

**Trial registration:**

ISRCTN registry, ISRCTN02484593. Registered on 7 January 2013.

**Electronic supplementary material:**

The online version of this article (doi:10.1186/s13063-017-2040-2) contains supplementary material, which is available to authorized users.

## Background

Depression is a major healthcare challenge in all parts of the world [1]. World Health Organization (WHO) data from 60 countries showed a 12 month prevalence of depressive episodes of 3.2% in patients without comorbidities and from 9.3 to 23.0% in those suffering from chronic healthcare problems [2]. The most commonly offered interventions include psychological treatment and antidepressant drugs. The WHO predicts that depression will become the leading burden of disease by 2030 and it has already become the number one reason for years lost to disability [1].

Some patients experience partial or no benefit of psychological interventions or pharmacotherapy, or have experienced or are concerned about side effects of antidepressants. Some of these consult homeopaths [3]. Homeopaths provide a combination of consultations and prescription of homeopathic medicines, guided by underlying basic homeopathy principles [4]. Treating “like with like” is the core principle, stating that substances causing certain symptoms in healthy persons, may cure those same symptoms in those who are ill. There is considerable debate around the use of homeopathy, which has a 12-month prevalence of 3.9% (Relton C, Cooper K, Viksveen P, Fibert P, Harris P, Thomas KJ. Prevalence of homeopathy use by the general population worldwide: a systematic review. Homeopathy. Forthcoming 2017 (under review)), in particular the highly diluted homeopathic medicines, which are produced through serial dilution and succussion (shaking) to reduce the risk of side effects. Depression is one of the conditions homeopaths are most commonly consulted for [5, 6]. An early systematic review found limited evidence available to assess the effectiveness and safety of homeopathy in depression [7]. Since then, results of two placebo-controlled trials have been published, suggesting homeopathic medicines may be non-inferior to an antidepressant and superior to placebo [8, 9]. Although placebo-controlled trials may be used to assess the efficacy of medications, they are less suitable to assess interventions in “real world” practice. Pragmatic trials assessing the effectiveness of the “whole treatment package” of interventions, including consultations and medication, may be of greater relevance to patients, clinicians and decision makers [10]. Goldacre suggested unblinded trials comparing visits to a homeopathy clinic against a General Practitioner’s (GP’s) treatment as usual might be the way forward for homeopathy research, as this is of more interest to patients [11]. This is the first pragmatic randomised controlled trial assessing the effectiveness of treatment provided by homeopaths for depressed patients.

The primary objective of this trial was to assess the clinical effectiveness of the offer of adjunctive treatment provided by homeopaths for patients with self-reported unipolar depression in addition to usual care, compared to usual care alone. The secondary objectives were to assess the effectiveness of received treatment. The acceptability and the safety of the intervention were also assessed.

## Methods

This pragmatic randomised controlled trial used the “cohort multiple randomised controlled trial” (cmRCT) design [12]. One third of all patients with self-reported depression who were eligible for participation were randomly selected to receive an offer of treatment provided by homeopaths as an adjunct to usual care. The remaining two thirds served as a control group receiving no offer of treatment and continuing treatment as usual. Participants were recruited through the Yorkshire Health Study, a population-based longitudinal observational cohort [13]. Ethics approval was obtained from the National Regional Ethics Service (REC reference 12/YH/0379) and the protocol was published prior to trial start (ISRCTN02484593) [14]. Data are reported in line with Additional file 1 [15].

### Recruitment

Patients in the Yorkshire Health Study cohort, who had been recruited through 43 GPs in South Yorkshire, had previously provided information on their state of health through a health questionnaire [13]. A mood and health screening questionnaire was sent to all those who had previously reported suffering from long-standing depression or feeling moderately or extremely anxious or depressed. All adults (age 18–85 years) who responded to this questionnaire and who fulfilled the inclusion criteria were included in the trial. Criteria for self-reported depression included scoring at least 10 points on the 9-item Patient Health Questionnaire (PHQ-9), including at least 2 points on question 1 (little interest/pleasure in doing things) or question 2 (feeling down, depressed or hopeless). PHQ-9 is based on the depression criteria of the *Diagnostic and Statistical Manual of Mental Disorders* (DSM-V). It has been compared to standard screening criteria and has been found to have a high degree of validity, reliability, sensitivity and specificity and it is considered a useful screening tool with a cutoff score for major depressive disorder of 10 points [16]. Exclusion criteria were self-reported: Alzheimer’s disease, bipolar disorder, organic brain damage, schizophrenia, schizoaffective disorders, other psychotic disorders, or antisocial personality disorder; having received treatment by a homeopath over the past 3 months; currently being involved in other health research; or being unable to understand study questionnaires and accompanying information due to reduced intellectual capacity, illiteracy or English language skills. Patients were recruited from 15 September 2013 to 7 February 2014 (randomisation 9 December 2013).

### Random selection and consent

One third of the patients who fulfilled the inclusion criteria were randomly selected to receive an offer of treatment by a homeopath. Random selection was carried out by a statistician not otherwise involved in the trial, using a computer software program, at three time points (9 December 2013, 14 January 2014 and 7 February 2014) depending on when patients returned their screening questionnaire. A simple randomisation process was applied (no block/stratification randomisation) at a 1:2 ratio. Only patients’ research ID numbers were provided for the random selection process. Patients in the cohort had consented to be contacted again and for their data to be used to assess the benefits of health interventions. Information about the intervention was sent to patients randomly selected to receive the offer of treatment and written informed consent was obtained from those accepting the offer, which included consenting to continue standard medication as prescribed by their GP/specialist.

### Blinding

Other than for the random selection process, no blinding was used due to the pragmatic nature of the research question “Is treatment by a homeopath effective for self-reported depression?” No blinding of assessment (statistical analyses) was used, as group allocation would become obvious due to the 1:2 randomisation ratio.

### Trial groups

The intervention was provided by seven homeopaths in South Yorkshire. As this was a pragmatic trial, practitioners were instructed to practise as they usually do, and no restrictions were put on the length/frequency of consultations or medicines they prescribed. They were, however, provided additional training and guidelines for reporting risks and adverse events. Treatment was offered to individual patients for up to 9 months, in three integrated health clinics in Barnsley, Doncaster and Sheffield, and in a medical centre in Rotherham. All patients were free to start or continue any other treatment during the trial.

### Outcomes

The primary outcome was the self-report PHQ-9 measure at 6 months. The PHQ-9 is sensitive to change and useful for assessing patients with various medical comorbidities [16]. The secondary outcome measures were PHQ-9 at 12 months; and the Generalized Anxiety Disorder (GAD-7) outcome measure at 6 and 12 months [17]. The GAD-7 has a high degree of reliability and validity for measuring anxiety in the general population and in heterogeneous groups of patients [18]. Both the PHQ-9 and GAD-7 are reported as continuous scores. Two secondary outcomes included in the study protocol were removed in order to reduce participant burden and one (body mass index (BMI)) was removed as there was no past research suggesting changes in scores could be expected. Patients were followed up in the time period from 9 December 2013 to 7 February 2015 (individual patients for 12 months).

### Sample size

The trial was designed to test the effectiveness of an offer of treatment provided by homeopaths as an adjunct to usual care (offer group), compared to usual care alone (no offer group). The effect size used for the sample size calculation was 0.35. Due to the lack of previous pragmatic trials in this field, the effect size was chosen under the assumption that a small to moderate effect size should be the minimum threshold level for recommending the intervention as an adjunct to usual care. The significance level (alpha error) was 0.05 and the power 80%. The questionnaire non-response rate was estimated to be 40%. Using an unequal randomisation ratio, this gave a sample size of 485 patients, with 162 in the offer group and 323 in the no offer group. A “rolling recruitment” method was used, including patients until a cutoff date for inclusion.

### Statistical methods

Intention-to-treat (ITT) analysis was used to assess the effectiveness of the offer of treatment provided by a homeopath as an adjunct to usual care (offer group), compared to usual care alone (no offer group), for all patients with analysable data. Patients remained in the group they were randomly allocated to, irrespective of whether they received the intervention or not. However, patients who were found post randomisation not to be eligible for participation (not fulfilling inclusion/exclusion criteria) were excluded from the analysis.

A general linear model (GLM) was used for the primary analysis, comparing the mean depression (PHQ-9) scores at 6 months post randomisation in the offer group and no offer groups, and controlling for baseline characteristics. Baseline characteristics were selected using linear regression as part of a hierarchical model testing the effect of 11 characteristics considered to potentially influence depression scores. The 11 characteristics tested were selected on the basis of the literature on depression research and the opinion of the trial researchers and practitioners. Characteristics with a *p* value of at least 0.2 were included in the model.

As the intervention was therapist-based, there could be clustering or correlation of patients’ outcomes and treatment offered by particular practitioners. To allow for this, mean PHQ-9 scores were assessed using generalised estimating equations (GEE) using an exchangeable correlation to estimate the regression coefficients. Participants in the control group were treated as one cluster. The exchangeable correlation assumed that participant outcomes within each cluster (practitioner group) had the same correlation.

At 12 months post randomisation, analysis of covariance (ANCOVA) was applied comparing mean outcomes in the offer and no offer groups, including outcomes at 6 and 12 months, and controlling for baseline characteristics. The influence of baseline characteristics was tested as for 6-month outcomes. Similar approaches were used to assess anxiety (GAD-7) scores at 6 and 12 months. All statistical exploratory tests were two-tailed with alpha set to 0.05. The 95% confidence interval (CI) for the difference in PHQ-9 scores was also calculated for all comparisons.

ITT analyses “water down” any potential effect of interventions in trials with low acceptance or compliance rates [19]. This is likely to be the case in trials using the cmRCT design, as patients in the offer group are only informed about the intervention after being included in the trial, and a proportion are likely to not accept the offer of treatment [20]. Moreover, trials in depression are known to have particular challenges with recruitment [21]. It was therefore probable that a significant proportion of patients would not take up the offer of treatment. Per-protocol analyses, which are often used by researchers to assess the effectiveness of treatment received, carry a high risk of bias. Instead, complier average causal effect (CACE) analyses have been recommended to take into account non-compliance in RCTs [22]. In CACE analyses, outcomes in patients who take up the offer of treatment are compared to outcomes in the no offer group of patients who would have taken up the offer had they received it. In this trial, instrumental variables (IV) analysis was used, as this type of CACE-analysis takes into account patients’ baseline characteristics.

### Missing data

Four approaches for dealing with missing data were used: no imputation for missing data, multiple imputation, regression imputation and last observation carried forward. Multiple imputation was selected as the primary method, as it was expected to provide more conservative results than no imputation for missing data. Little’s missing completely at random (MCAR) test did not suggest any systematic patterns in missing data.

### Effect size

The standardized effect size was calculated by dividing the difference in the change of the estimated marginal mean depression (PHQ-9) scores between the offer and no offer groups from baseline to 6 months, by the pooled standard deviation of baseline PHQ-9 scores [23]. Similar approaches were used for secondary outcomes.

### Assessment of acceptability and safety

The acceptability of the intervention was assessed by calculating the acceptance rate in patients receiving the offer of treatment. The safety of treatment was assessed by considering the number and nature of adverse events and potential risks (suicide, self-harm, causing harm to others, significant mental deterioration) reported by patients and practitioners and identified through qualitative interviews carried out by the first researcher. Two researchers, independently of each other, assessed the severity and causality of all reported adverse events. For most adverse events (19/24) the researchers’ assessments corresponded. All discrepancies in assessments were resolved through discussion. The severity of adverse events was assessed using the common terminology criteria for adverse events. The WHO and Uppsala Monitoring Centre guidelines were used for assessment of causality of adverse events.

## Results

### Recruitment, random selection and questionnaire response

**Fig. 1:**
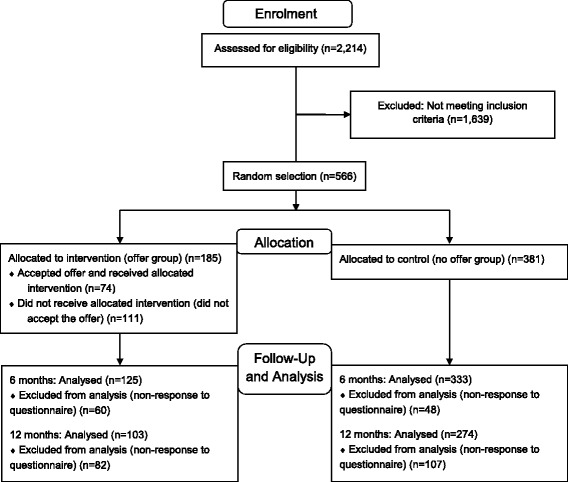
Consolidated standards of reporting trials (CONSORT) flow-diagram: recruitment, randomisation and flow of patients

At the time of recruitment, the Yorkshire Health Study cohort included 22,179 patients who had consented to being contacted again. A total of 5740 patients who had previously reported long-standing depression or feeling moderately or extremely anxious or depressed were sent a screening questionnaire (15 September 2013). Out of these, 2214 (38.6%) returned a completed questionnaire (7 February 2014), with 566 fulfilling the inclusion criteria and 185 randomly selected to receive the offer of treatment (Fig. 1).

At 6 months, 458 patients (81%) returned a follow-up questionnaire, with 125 (68%) in the offer group and 333 (87%) in the no offer group. Among those in the offer group who took up the offer of treatment, 88% responded (n = 65/74), compared to only 54% of those who did not take up the offer (n = 60/111).

At 12 months, 377 patients (67%) returned a completed questionnaire, with 72% (n = 274) in the no offer group and 56% (n = 103) in the offer group. As for the 6-month questionnaires, the proportion of responders in the offer group was higher among patients who took up the offer of treatment (n = 58, 78%) than among those who did not (n = 45, 41%).

### Comparability of questionnaire responders and non-responders

In order to consider the appropriateness of comparing outcomes of responders in the offer and no offer groups, which had different response rates, the comparability of baseline characteristics of responders and non-responders to the 6-month and 12-month questionnaires in the offer and no offer groups was assessed using a multiple linear regression model.

No evidence was found suggesting there were significant differences in depression or anxiety scores at baseline between responders and non-responders to the 6-month and 12-month questionnaires in the offer and the no offer groups (data not shown). At 6 months, statistically significant differences (≤0.05) were found in 5 of 20 baseline characteristics (age, employment status, deprivation score, existence of more than three long-standing conditions and the proportion of patients reporting whether they were using antidepressants), but none were considered likely to significantly influence 6-month outcomes. At 12 months, the only statistically significant differences in baseline characteristics included lower likelihood of non-responders in the offer group reporting the time of onset of their depression and lower likelihood of reporting whether they had used antidepressants in the past. It was considered to be unlikely that this would influence 12-month outcomes. Furthermore, no statistically significant differences in baseline characteristics were found between patients in the offer group who received the intervention and those who did not (data not shown).

Statistical analyses of the outcomes in the offer and no offer groups at 6 and 12 months could therefore be carried out with limited risk of significant influence of any known potential confounding due to differences in the response rates.

### Baseline characteristics

The trial included patients of all age groups ranging from 18 to 85 years (mean 55 years, SD 15). The majority were female (61%) and of British ethnic origin (96%). Fewer were employed (38%) than not (56%), and over 60% were among the most (42%) and second most (19%) deprived quintiles of patients. Depression, anxiety and other baseline characteristics were comparable in the offer and no offer groups (Tables 1, 2 and 3).

**Table 1:** Baseline characteristics

Baseline characteristics	Offer group (*n* = 185)	No offer group (*n* = 381)	Total (*n* = 566)
Age (years) (mean, SD)	55.8 (15.0)	53.9 (14.4)	54.5 (14.6)
Female	114 (61.6)	229 (60.1)	343 (60.6)
Children ≤2 years	9 (4.9)	14 (3.9)	24 (4.2)
Pregnant	0 (0.0)	4 (1.0)	4 (0.7)
Ethnicity:			
British	179 (96.8)	364 (95.5)	543 (95.9)
Non-British	6 (3.2)	15 (3.9)	21 (3.7)
Unknown	0 (0.0)	2 (0.5)	2 (0.4)
Employed:			
Yes	64 (34.6)	152 (39.9)	216 (38.2)
No	111 (60.0)	209 (54.9)	320 (56.5)
Unknown	10 (5.4)	20 (5.2)	30 (5.3)
Index of Multiple Deprivation (IMD) quintile:			
1 (least deprived)	11 (5.9)	21 (5.5)	32 (5.7)
2	34 (18.4)	76 (19.9)	110 (19.4)
3	26 (14.1)	55 (14.4)	81 (14.3)
4	34 (18.4)	73 (19.2)	107 (18.9)
5 (most deprived)	80 (43.2)	156 (40.9)	236 (41.7)

Figures are frequencies (percentages) unless stated otherwise

**Table 2:** Baseline health measures

Baseline health measures	Offer group (n = 185)	No offer group (n = 381)	Total (n = 566)
PHQ-9 score (mean, SD)	16.9 (4.5)	17.0 (4.6)	17.0 (4.6)
PHQ-9 category:			
Moderate (10–14 points)	64 (34.6)	133 (34.9)	197 (34.8)
Moderately severe (15–19 points)	69 (37.3)	133 (34.9)	202 (35.7)
Severe (20–27 points)	52 (28.1)	115 (30.2)	167 (29.5)
Depression onset:			
Acute (<3 months)	2 (1.1)	4 (1.0)	6 (1.1)
Sub-acute (3 months– < 1 year)	5 (2.7)	14 (3.7)	19 (3.4)
Chronic (short) (1– < 2 years)	12 (6.5)	39 (10.2)	51 (9.0)
Chronic (long) (2– < 5 years)	10 (5.4)	31 (8.1)	41 (7.2)
Chronic (very long) (5 years +)	89 (48.1)	185 (48.6)	274 (48.4)
Chronic (any)	111 (60.0)	255 (66.9)	366 (64.7)
Unknown	67 (36.2)	108 (28.3)	175 (30.9)
Depression episode onset:			
Acute (<3 months)	10 (5.4)	24 (6.3)	34 (6.0)
Sub-acute (3 months– < 1 year)	8 (4.3)	25 (6.6)	33 (5.8)
Periodic	3 (1.6)	14 (3.7)	17 (3.0)
Chronic (short) (1– < 2 years)	9 (4.9)	13 (3.4)	22 (3.9)
Chronic (long) (2– < 5 years)	8 (4.3)	11 (2.9)	19 (3.4)
Chronic (very long) (5 years+)	9 (4.9)	13 (3.4)	22 (3.9)
Chronic (unspecified)	30 (16.2)	46 (12.1)	76 (13.4)
Chronic (any)	56 (30.3)	83 (21.8)	139 (24.6)
None (patient’s opinion)	1 (0.5)	3 (0.8)	4 (0.7)
Unknown	107 (57.8)	232 (60.9)	343 (60.6)
GAD-7 score (mean, SD)	13.4 (4.8)	13.8 (4.8)	13.7 (4.8)
GAD-7 category:			
Normal (0–4 points)	6 (3.2)	14 (3.7)	20 (3.5)
Mild (5–9 points)	32 (17.3)	59 (15.5)	91 (16.1)
Moderate (10–14 points)	68 (36.8)	126 (33.1)	194 (34.3)
Severe (15–21 points)	78 (42.2)	181 (47.5)	259 (45.8)
Unknown	1 (0.5)	1 (0.3)	2 (0.4)
BMI score (mean, SD)	28.3 (6.6)	28.1 (6.6)	28.2 (6.6)
BMI category:			
Underweight (<18.5)	3 (1.6)	8 (2.1)	11 (1.9)
Healthy weight (18.5–24.9)	58 (31.4)	129 (33.9)	187 (33.0)
Overweight (25.0–29.9)	37 (20.0)	98 (25.7)	135 (23.9)
Obese (30.0–39.9)	51 (27.6)	98 (25.7)	149 (26.3)
Morbidly obese (40.0+)	11 (5.9)	20 (5.2)	31 (5.5)
Unknown	25 (13.5)	28 (7.3)	53 (9.4)
Long-standing conditions (mean, SD)	3.0 (1.8)	3.0 (1.8)	3.0 (1.8)
Long-standing conditions:			
Yes	165 (89.2)	350 (91.9)	515 (91.0)
No	17 (9.2)	25 (6.6)	42 (7.4)
Unknown	3 (1.6)	6 (1.6)	9 (1.6)
Alcohol (units last week) (mean, SD)	7.0 (16.4)	7.7 (15.0)	7.4 (15.5)

Figures are frequencies (percentages) unless stated otherwise. *PHQ* Patient Health Questionnaire, *GAD* Generalised Anxiety Disorder, *BMI* body mass index

**Table 3:** Baseline medication and treatment

Baseline health measures	Offer group (n = 185)	No offer group (n = 381)	Total (n = 566)
Antidepressant use (current):			
Yes	81 (43.8)	155 (40.7)	236 (41.7)
No	0 (0.0)	1 (0.3)	1 (0.2)
Unknown	104 (56.2)	255 (59.1)	329 (58.1)
Antidepressant use (past):			
Yes	103 (55.7)	234 (61.4)	337 (59.5)
No	53 (28.6)	124 (32.5)	177 (31.3)
Unknown	29 (15.7)	23 (6.0)	52 (9.2)
Medication (current, all types) (mean, SD)	4.7 (4.0)	4.7 (3.9)	4.7 (3.9)
Visits last 3 months (mean, SD):			
Hospital (accident & emergency)	0.5 (1.6)	0.4 (0.6)	0.4 (1.0)
Hospital (day case)	0.5 (1.6)	0.4 (0.9)	0.4 (1.2)
Hospital (out-patient)	1.4 (2.5)	1.2 (2.0)	1.3 (2.2)
Hospital (in-patient nights)	0.6 (2.2)	0.8 (2.9)	0.8 (2.7)
General Practitioner	3.0 (5.6)	2.5 (2.4)	2.6 (3.8)
Nurse	1.9 (6.2)	1.4 (2.4)	1.6 (4.0)
Physiotherapist	1.0 (2.6)	0.6 (1.8)	0.8 (2.1)
Other^a^	1.9 (9.3)	1.2 (3.8)	1.4 (6.2)

Figures are frequencies (percentages) unless stated otherwise. ^a^Summary including dietitian, midwife, mental health worker, psychotherapist, counsellor, care worker, social worker, health visitor, community health champion, health trainer, acupuncturist, chiropractor, herbalist, osteopath (homeopath was 0 for both groups, as prior use was an exclusion criterion)

The patients’ mean depression score was 17.0 (SD 4.6), measured on the 0–27 point self-report PHQ-9. A slightly larger proportion suffered from moderate (35%) and moderately severe (36%) depression, than severe (30%) depression. Over 90% of patients reporting on when their depression first started (n = 391, 69%), could be categorised as suffering from chronic self-reported depression (lasting a minimum of 1 year), and 70% had suffered from depression for at least 5 years. The onset of the current depression episode was only reported by 40% (n = 227), and among these patients over 60% suffered from a chronic depression episode (minimum of 1 year).

The average anxiety score, measured using the self-report GAD-7 outcome measure (range 0–21), was 13.7 (SD 4.8), with 80% suffering from moderate (34%) or severe (46%) anxiety. More than 90% reported suffering from at least one long-standing condition, with an average of 3.0 (SD 1.8) long-standing conditions. The mean BMI score was 28.2 (SD 6.6), with 56% being overweight (24%), obese (26%) or morbidly obese (6%).

Four in ten patients were taking antidepressant drugs, and 60% had done so in the past. On average, patients were taking 4.7 (SD 3.9) different drugs. The most commonly used healthcare practitioners/services over the past 3 months were GPs (mean 2.6 consultations, SD 3.8), nurses (1.6, SD 4.0) or hospital outpatient services (1.3, SD 2.2) (Table 3). The use of mental health workers (0.7, SD 2.2), counsellors (0.7, SD 2.6) and psychotherapists (0.4, SD 1.5) was less common (data not shown).

Higher depression scores were significantly correlated with higher anxiety scores (*p* < 0.01), greater numbers of long-standing conditions (*p* < 0.001) and higher BMI scores (p = 0.010). There was no correlation between depression scores and current antidepressant use (*p* = 0.511), but there was correlation between depression scores and greater use of GPs (*p* = 0.003), mental health workers (*p* = 0.001) and counsellors (*p* = 0.009).

Although patients in our trial were more likely to suffer from chronic depression and the non-British ethnic population was underrepresented, baseline characteristics were overall comparable to other studies. As in our trial, other studies have shown that depression is more common in women [24] and in populations with higher unemployment [25] and lower socio-economic status [26]. Anxiety is the most common comorbidity [27] and it is also correlated witho obesity [28].

### The intervention

Over the 12 months of the trial, the 74 patients who took up the offer of treatment received a total of 490 consultations with a homeopath, with a median of 7.5 (interquartile range 4.8–9.0) consultations over 6.5 months (IQR 4.1–8.9). Consultations lasted a median of 57 minutes (IQR 48–65) and 526 prescriptions (median per patient 7.0, IQR 4.0–9.3) of 68 different homeopathic medicines were made. In 18% of consultations, patients received additional advice, such as recommendations to make dietary changes (e.g. reduce sugar/fat intake), to consult with other practitioners (mostly GPs), initiate self-care measures (e.g. stress-reducing exercises), or use various other products (e.g. supplements or herbal teas). No patient was recommended St. John’s Wort, a herb commonly used in the treatment of depression.

### Acceptability

Of 185 patients, 74 (40%) took up the offer of treatment and had at least one consultation with a homeopath. Out of these patients, 90.5% (n = 67) had more than one consultation, 75.7% (n = 56) had 5–12 consultations, whereas 9.5% (n = 7) only had one consultation.

### Effectiveness in the offer group

#### Depression outcomes and estimation

The primary analysis showed a mean between-group difference in depression (PHQ-9) scores in favour of the offer group at 6 months of 1.4 points (95% CI 0.2, 2.5, *p* = 0.019), suggesting a small standardized effect size in the offer group (Cohen’s *d* = 0.30). Results were maintained at 12 months (ANCOVA: mean difference 1.4 points, 95% CI 0.3, 2.5, *p* = 0.015, Cohen’s *d* = 0.30). Secondary analyses at 6 and 12 months were comparable to the main analysis, although 3 out of 31 tests were not statistically significant (details in Additional files).

#### Anxiety outcomes and estimation

The primary analysis of the anxiety (GAD-7) outcome measure showed a mean between-group difference in favour of the offer group at 6 months of 1.5 points (95% CI 0.5, 2.5, *p* = 0.003), with a small standardized effect size in the offer group (Cohen’s *d* = 0.33). Results were maintained at 12 months (ANCOVA: mean difference 1.6 points, 95% CI 0.6, 2.6, *p* = 0.002, Cohen’s *d* = 0.33). For anxiety, secondary analyses varied somewhat more than for depression outcomes, ranging from 1.0 to 1.5 points in favour of the offer group at 6 months, with non-statistically significant results for 5 out of 15 tests, and from 1.4 to 2.1 points at 12 months, with non-statistically significant results for 6 out of 16 tests (details in Additional files).

### Effectiveness of treatment received

#### Depression outcomes and estimation

The primary IV analysis showed a mean between-group difference in depression (PHQ-9) scores at 6 months of 2.6 points (95% CI 0.5, 4.7, *p* = 0.018) in favour of patients who received treatment by a homeopath, suggesting a moderate standardized effect size (Cohen’s *d* = 0.57). Results were maintained at 12 months (mean difference 2.4 points, 95% CI 0.9, 4.0, *p* = 0.002, Cohen’s *d* = 0.53). Results of secondary analyses at 6 and 12 months were comparable to the main analyses and all results were statistically significant (details in Additional files).

#### Anxiety outcomes and estimation

The primary IV analysis showed a mean between-group difference in anxiety (GAD-7) scores at 6 months of 2.8 points (95% CI 0.9, 4.8, *p* = 0.004) in favour of patients who received treatment by a homeopath, suggesting a moderate standardized effect size (Cohen’s *d* = 0.61). Results were maintained at 12 months (mean difference 2.8 points, 95% CI 1.4, 4.2, p = 0.000, Cohen’s *d* = 0.59). Results of secondary analyses at 6 and 12 months were comparable to the main analyses, although one of the 16 tests was not statistically significant (details in Additional files).

### Adverse events and risks

Two researchers categorised 14 adverse events in 12 patients (16.2% of all treated patients in the offer group) as certain (n = 1), probable/likely (n = 7), or possibly (n = 6) to be related to the intervention. Most adverse events were mild (n = 11). Two events (in one patient) were categorised as severe, as the patient described them as “severe headache” and “severe pain in chest, back, neck and jaw”. The events were transient and were considered by the researchers to be only possibly related to the intervention as medical/hospital exams did not reveal pathological conditions and the symptoms did not return on repeating the homeopathic medication (re-challenge). An aggravation of chronic pain lasting for 3 weeks in another patient was categorised as a moderate adverse event, but only possibly related to the intervention, as it did not return on re-challenge. The only adverse event considered to be certain to be related to the intervention was the bad odour/taste of a homeopathic medication (confirmed by the homeopath). The manufacturer suggested it might be due to the medication being past its expiry date. Another 10 adverse events were unclassified (n = 1) or considered unlikely (n = 9) to be related to the intervention.

In addition, potential risk was considered in 58 patients (10.2%), with 36 in the no offer group (9.4%) and 22 in the offer group (11.9%). Most patients (n = 55) were considered to be at potential risk as they reported not using antidepressants and indicating on a follow-up questionnaire that they had thoughts nearly every day of being better off dead or of hurting themselves. Patients were contacted by telephone for further risk assessment and their GPs were notified when patients could not be reached. No serious adverse events are known to have occurred in any of these patients.

## Discussion

This was the first pragmatic randomised controlled trial assessing the effectiveness of treatment provided by homeopaths for depressed patients. The statistical analysis plan for the trial stated that a standardized effect size would be used for assessing the clinical importance of the results and these results suggest a small clinical effect of the offer of treatment and a moderate effect of treatment received by patients. However, there is some evidence to suggest that the threshold level for the minimal clinically important difference (MCID) for PHQ-9 is a reduction of 5 points [29], which is more than the score detected in this trial. Although wide confidence intervals preclude any firm conclusions from being drawn, these results warrant further research into this intervention for depressed patients for three reasons.

First, existing interventions for depression are also mostly associated with small effect sizes, e.g. anti-depressants and psychological interventions with small to moderate effect sizes [30, 31]. Although some trials of psychotherapeutic interventions suggest moderate to large effect sizes, most have unknown or high risk of bias, and only smaller effects are seen when controlling for unpublished trials or assessing higher quality studies with larger sample sizes [30, 32].

Second, the results of this trial must be seen in light of the fact that the majority of patients suffered from long-standing depression and several comorbidities, and they are therefore harder to treat and have a poorer prognosis [33].

Third, although this trial was not powered to detect side-effects, no evidence suggested the treatment was unsafe. Antidepressants are commonly associated with risk of unwanted side-effects, some of which may be severe and under-reported, and some argue that the harms of antidepressants may outweigh the benefits [34–36].

The trial over-recruited within 5 months, a good result when seen in light of the fact that trials often struggle to reach recruitment goals [37]. It was anticipated that a large proportion of patients would decline the offer of treatment as this was not a treatment-seeking population – thus, patients were unaware of the possibility of being offered the intervention before trial start in this trial within a cohort design [20]. Potential non-acceptance of an offer of treatment is a specific feature of the cmRCT design that warrants particular attention when interpreting trial results. Regular ITT analyses represent the effect of an “offer” of treatment, although we do not suggest there is an effect simply of being offered the intervention. ITT analyses will “water down” any potential effect of interventions in cmRCT trials with low acceptance or compliance rates. Therefore, IV analysis should be applied to test the effectiveness of the received intervention. Compared to “regular” RCTs, the use of the cmRCT design provides the additional benefit of testing the acceptability of the intervention. Treatment uptake in this particular trial was good, given that this was not a clinical-treatment-seeking population and the controversy surrounding homeopathy in the UK over the past few years.

This was the first full trial of any intervention using the cmRCT design. The benefits and challenges of using this design of trials within a cohort (http://www.twics.global) included full and fast recruitment, low attrition and recruitment of a population reasonably similar to the population of patients who self-report chronic depression (Viksveen P, Relton C, Nicholl J. Benefits and challenges of using the “cohort multiple randomised controlled trial” design for testing an intervention for depression. Trials. Forthcoming 2017 (accepted)).

A limitation of this trial was a lower 6-month and 12-month questionnaire response rate in patients who were randomly selected to be offered the intervention, but who did not take up the offer. There was, however, no evidence to suggest that any baseline characteristics were likely to significantly affect outcomes, so statistical analyses of outcomes could be carried out with limited risk of significant influence of known potential confounding due to differences in response rates. Another limitation of this research was the lack of blinding of the researcher who collected questionnaire responses and carried out statistical analyses. Questionnaires were, however, completed by patients at home, thereby avoiding any undue research influence on patients, and data management was checked by two other researchers.

This trial was not designed to answer the question of whether or not homeopathic medicines are effective in treating depression, but whether treatment provided by homeopaths for patients with self-reported depression is effective. This treatment typically includes an initial session lasting from one to two hours involving taking history, clinical examination, discussion of treatment options, and prescription of homeopathic medicines, followed by up to ten sessions at monthly intervals to review progress and adjust treatments. The pragmatic nature of this trial; with recruitment of patients from a large population-based health cohort, wide inclusion criteria and encouraging practitioners to practise as they normally do, increased the generalisability of results to the general population of patients suffering from long-standing self-reported depression who take up an offer of treatment provided by a homeopath. Research questions that need further consideration if results of this trial were to be reproduced include an understanding of the mechanism of action, the effect of homeopathic medicines and of the homeopath-patient interaction, and indirect effects that may influence outcomes (e.g. changes in patients’ medication).

## Conclusion

An offer of treatment provided by homeopaths for patients with self-reported depression was associated with a small treatment effect over a time period of 6 to 12 months, whereas a moderate effect was found in patients who received treatment. However, wide confidence intervals preclude any firm conclusions from being drawn. Further research should be conducted in order to see if these findings can be replicated, and to determine the safety of this adjunctive treatment.

## Additional files

Additional file 1: CONSORT 2010 checklist of information to include when reporting a randomised trial. (DOC 219 kb)

Additional file 2: Table S1. Depression outcomes at 6 and 12 months. Intention-to-treat analysis of the offer of treatment. (DOCX 15 kb)

Additional file 3: Table S2. Anxiety outcomes at 6 and 12 months. Intention-to-treat analysis of the offer of treatment. (DOCX 15 kb)

Additional file 4: Table S3. Depression outcomes at 6 and 12 months. Instrumental variables analysis of treatment received. (DOCX 14 kb)

Additional file 5: Table S4. Anxiety outcomes at 6 and 12 months. Instrumental variables analysis of treatment received. (DOCX 14 kb)

## Abbreviations

ANCOVA: Analysis of covariance
BMI: Body mass index
CACE: Complier average causal effect
CI: Confidence interval
cmRCT: Cohort multiple randomised controlled trial
DSM-V: *Diagnostic and Statistical Manual of Mental Disorders*
GAD-7: Generalised anxiety disorder
GEE: Generalised estimating equations
GLM: General linear model
GP: General Practitioner
IMD: Index of Multiple Deprivation
IQR: Interquartile range
ITT: Intention-to-treat
IV: Instrumental variables
MCAR: Missing completely at random
MCID: Minimal clinically important difference
PHQ-9: Patient Health Questionnaire
REC: Regional Ethics Committee
SD: Standard deviation
WHO: World Health Organization
