# Supplementary material for: Depressed patients treated by homeopaths: a randomised controlled trial using the “cohort multiple randomised controlled trial” (cmRCT) design
Source: Trials. 2017 Jun 30;18:299. doi: 10.1186/s13063-017-2040-2 (PMC5493124; doi:10.1186/s13063-017-2040-2)
Supplement: Supplementary file 2 — Depression outcomes at 6 and 12 months. Intention-to-treat analysis of the offer of treatment. (DOCX 15 kb) [file 13063_2017_2040_MOESM2_ESM.docx]

| **Additional file 2: Table S1. Depression outcomes at 6 and 12 months. Intention-to-treat analysis of the offer of treatment.** | | |
| --- | --- | --- |
|  | **Mean between group difference^a^ (95% CI),**  **p-value, standardised effect size** | |
| **Analysis** | **6 months (n=458)*** | **6&12 months (n=377) **** |
| **Primary analysis** |  |  |
| GLM with MI for missing data | 1.4 (0.2, 2.5), 0.019, 0.30 | 1.4 (0.3, 2.5), 0.015, 0.30 |
| **Secondary analyses** |  |  |
| Controlling for baseline PHQ-9 score |  |  |
| GLM with RI for missing data | 1.4 (0.3, 2.5), 0.011, 0.31 | 1.5 (0.4, 2.7), 0.009, 0.33 |
| GLM with LOCF for missing data | 1.3 (0.2, 2.4), 0.018, 0.29 | 1.3 (0.2, 2.4), 0.025, 0.27 |
| GLM with no imputation for missing data | 1.6 (0.5, 2.8), 0.005, 0.36 | 1.5 (0.4, 2.7), 0.010, 0.33 |
| GEE with MI for missing data | 1.3 (0.2, 2.4), 0.026, 0.28 | 1.7 (-0.1, 3.5), 0.058, 0.37 |
| GEE with RI for missing data | 1.4 (0.3, 2.4), 0.010, 0.30 | 1.9 (0.4, 3.4), 0.016, 0.41 |
| GEE with LOCF for missing data | 1.3 (0.1, 2.4), 0.028, 0.28 | 1.9 (0.3, 3.4), 0.020, 0.40 |
| GEE with no imputation for missing data | 1.6 (0.5, 2.6), 0.003, 0.34 | 1.9 (0.6, 3.3), 0.006, 0.41 |
| Controlling for multiple baseline characteristics *** |  |  |
| GLM with MI for missing data | 1.2 (0.1, 2.4), 0.032, 0.27 | 1.4 (0.3, 2.5), 0.010, 0.31 |
| GLM with RI for missing data | 1.3 (0.2, 2.3), 0.021, 0.28 | 1.6 (0.5, 2.7), 0.005, 0.35 |
| GLM with LOCF for missing data | 1.2 (0.1, 2.3), 0.032, 0.27 | 1.3 (0.2, 2.4), 0.017, 0.28 |
| GLM with no imputation for missing data | 1.5 (0.4, 3.7), 0.010, 0.33 | 1.6 (0.5, 2.8), 0.006, 0.35 |
| GEE with MI for missing data | 1.2 (-0.0, 2.3), 0.051, 0.26 | 1.8 (0.1, 3.5), 0.034, 0.39 |
| GEE with RI for missing data | 1.2 (0.2, 2.3), 0.025, 0.27 | 2.0 (0.5, 3.4), 0.007, 0.42 |
| GEE with LOCF for missing data | 1.2 (-0.0, 2.3), 0.057, 0.25 | 1.9 (0.5, 3.4), 0.009, 0.42 |
| GEE with no imputation for missing data | 1.4 (0.4, 2.5), 0.006, 0.32 | 2.0 (0.7, 3.2), 0.002, 0.43 |
| a All differences in favour of the offer group (lower PHQ-9 scores). * Primary end-point. ** ANCOVA tests including 6 & 12 month data, except GEE analyses with 12 months data only. *** At 6 months: Controlling for baseline PHQ-9 scores, current antidepressant use, more than 3 long-standing conditions, gender. At 12 months: Controlling for baseline PHQ-9 scores, current antidepressant use, deprivation quintile, gender. | | |
