# Supplementary material for: Depressed patients treated by homeopaths: a randomised controlled trial using the “cohort multiple randomised controlled trial” (cmRCT) design
Source: Trials. 2017 Jun 30;18:299. doi: 10.1186/s13063-017-2040-2 (PMC5493124; doi:10.1186/s13063-017-2040-2)
Supplement: Supplementary file 3 — Anxiety outcomes at 6 and 12 months. Intention-to-treat analysis of the offer of treatment. (DOCX 15 kb) [file 13063_2017_2040_MOESM3_ESM.docx]

| **Additional file 3: Table S2. Anxiety outcomes at 6 and 12 months. Intention-to-treat analysis of the offer of treatment.** | | |
| --- | --- | --- |
|  | **Mean between group difference^a^ (95% CI),**  **p-value, standardised effect size** | |
| **Analysis** | **6 months (n=456)*** | **6&12 months (n=377)**** |
| **Primary analysis** |  |  |
| GLM with MI for missing data | 1.5 (0.5, 2.5), 0.003, 0.33 | 1.6 (0.6, 2.6), 0.002, 0.33 |
| **Secondary analyses** |  |  |
| Controlling for baseline GAD-7 score |  |  |
| GLM with RI for missing data | 1.4 (0.4, 2.4), 0.005, 0.30 | 1.5 (0.4, 2.7), 0.009, 0.33 |
| GLM with LOCF for missing data | 1.4 (0.5, 2.4), 0.004, 0.31 | 1.5 (0.5, 2.4), 0.004, 0.31 |
| GLM with no imputation for missing data | 1.2 (0.2, 2.3), 0.023, 0.26 | 1.4 (0.4, 2.4), 0.004, 0.30 |
| GEE with MI for missing data | 1.5 (0.1, 2.9), 0.042, 0.32 | 2.1 (-0.1, 4.3), 0.066, 0.45 |
| GEE with RI for missing data | 1.3 (0.2, 2.5), 0.022, 0.29 | 1.8 (-0.2, 3.9), 0.082, 0.39 |
| GEE with LOCF for missing data | 1.4 (0.1, 2.7), 0.035, 0.30 | 2.1 (0.0, 4.1), 0.047, 0.44 |
| GEE with no imputation for missing data | 1.2 (-0.3, 2.6), 0.108, 0.25 | 1.9 (-0.2, 4.0), 0.077, 0.39 |
| Controlling for multiple baseline characteristics *** |  |  |
| GLM with MI for missing data | 1.2 (0.2, 2.2), 0.019, 0.26 | 1.5 (0.6, 2.5), 0.001, 0.33 |
| GLM with RI for missing data | 1.2 (0.2, 2.2), 0.015, 0.26 | 1.4 (0.5, 2.3), 0.004, 0.29 |
| GLM with LOCF for missing data | 1.1 (0.1, 2.1), 0.027, 0.24 | 1.4 (0.4, 2.3), 0.004, 0.29 |
| GLM with no imputation for missing data | 1.0 (-0.0, 2.1), 0.060, 0.21 | 1.7 (0.7, 2.7), 0.001, 0.35 |
| GEE with MI for missing data | 1.2 (-0.2, 2.5), 0.092, 0.25 | 2.0 (-0.0, 4.1), 0.051, 0.43 |
| GEE with RI for missing data | 1.2 (0.0, 2.3), 0.045, 0.25 | 1.8 (0.1, 3.7), 0.069, 0.37 |
| GEE with LOCF for missing data | 1.1 (-0.2, 2.3), 0.090, 0.23 | 2.0 (0.2, 3.8), 0.031, 0.42 |
| GEE with no imputation for missing data | 1.0 (-0.4, 2.4), 0.172, 0.20 | 1.9 (-0.0, 3.8), 0.051, 0.39 |
| a All differences in favour of the offer group (lower GAD-7 scores). * Primary end-point. ** ANCOVA tests including 6 & 12 month data, except GEE analyses with 12 months data only. *** At 6 months: Baseline GAD-7 scores, current antidepressant use, more than 3 long-standing conditions, PHQ-9 scores, employment status, gender. At 12 months: Baseline GAD-7 scores, current antidepressant use, PHQ-9 scores, deprivation quintile. | | |
