# Supplementary material for: Depressed patients treated by homeopaths: a randomised controlled trial using the “cohort multiple randomised controlled trial” (cmRCT) design
Source: Trials. 2017 Jun 30;18:299. doi: 10.1186/s13063-017-2040-2 (PMC5493124; doi:10.1186/s13063-017-2040-2)
Supplement: Supplementary file 4 — Depression outcomes at 6 and 12 months. Instrumental variables analysis of treatment received. (DOCX 14 kb) [file 13063_2017_2040_MOESM4_ESM.docx]

| **Additional file 4: Table S3. Depression outcomes at 6 and 12 months. Instrumental Variables analysis of treatment received.** | | |
| --- | --- | --- |
|  | **Mean between group difference^a^ (95% CI),**  **p-value, standardised effect size** | |
| **Analysis** | **6 months (n=458) *** | **6 & 12 months (n=377)** |
| **Primary analysis** |  |  |
| GLM with MI for missing data | 2.6 (0.5, 4.7), 0.018, 0.57 | 2.4 (0.9, 4.0), 0.002, 0.53 |
| **Secondary analyses** |  |  |
| Controlling for baseline PHQ-9 score |  |  |
| GLM with RI for missing data | 2.8 (0.7, 4.9), 0.008, 0.61 | 2.8 (1.2, 4.4), 0.001, 0.60 |
| GLM with LOCF for missing data | 2.6 (0.5, 4.7), 0.017, 0.56 | 2.2 (0.7, 3.8), 0.005, 0.48 |
| GLM with no imputation for missing data | 2.9 (0.7, 5.0), 0.010, 0.63 | 2.9 (1.3, 4.5), 0.000, 0.62 |
| Controlling for multiple baseline characteristics ** |  |  |
| GLM with MI for missing data | 2.3 (0.2, 4.5), 0.031, 0.52 | 2.5 (1.0, 4.1), 0.001, 0.55 |
| GLM with RI for missing data | 2.5 (0.5, 4.6), 0.015, 0.55 | 2.9 (1.4, 4.5), 0.000, 0.63 |
| GLM with LOCF for missing data | 2.3 (0.2, 4.4), 0.031, 0.50 | 2.3 (0.8, 3.9), 0.003, 0.50 |
| GLM with no imputation for missing data | 2.6 (0.5, 4.8), 0.018, 0.57 | 3.0 (1.4, 4.6), 0.000, 0.65 |
| a All differences in favour of treatment received (lower PHQ-9 scores). * Primary end-point. ** At 6 months: Baseline PHQ-9 scores, current antidepressant use, more than 3 long-standing conditions, gender. At 12 months: Baseline PHQ-9 scores, current antidepressant use, deprivation quintile, gender. | | |
